# TNF‐α augments CXCR2 and CXCR3 to promote progression of renal cell carcinoma

**DOI:** 10.1111/jcmm.12890

**Published:** 2016-06-14

**Authors:** Kuang‐Hui Sun, Guang‐Huan Sun, Yi‐Ching Wu, Bai‐Jiun Ko, Hui‐Tzu Hsu, Sheng‐Tang Wu

**Affiliations:** ^1^ Department of Biotechnology and Laboratory Science in MedicineNational Yang‐Ming UniversityTaipeiTaiwan; ^2^ Department of Education and ResearchTaipei City HospitalTaipeiTaiwan; ^3^ Division of UrologyDepartment of SurgeryTri‐Service General Hospital and National Defense Medical CenterTaipeiTaiwan

**Keywords:** kidney cancer, tumour microenvironment, chemokine, patient prognosis

## Abstract

Within the tumour microenvironment, a complex network of chemokines and their receptors affects the initiation and progression of tumours. The higher levels of tumour necrosis factor‐alpha (TNF‐α) are associated with tumour progression and an anti‐TNF‐α monoclonal antibody has been used successfully to treat patients with renal cell carcinoma (RCC). However, the role of chemokines and their receptors in the TNF‐α‐promoted progression of RCC remains unclear. In this study, TNF‐α was found to enhance the migration, invasion and epithelial‐mesenchymal transition (EMT) of RCC cells. To further investigate the molecular mechanism of TNF‐α on the progression of RCC, reverse transcription and quantitative PCR was used to screen chemokines and chemokine receptors that were associated with tumorigenesis. The results showed that TNF‐α significantly increased the expressions of CXCR2 and CXCR3 and their related ligands in RCC cells. Subsequently, we used a lentiviral shRNA system to knockdown the expression of CXCR2 and/or CXCR3 in RCC cells. CXCR2 and CXCR3 silencing inhibited the induction of Slug and ZEB‐1 with TNF‐α treatment of RCC cells. In addition, the knockdown of both CXCR2 and CXCR3 resulted in a greater decrease in cell migration, invasion and clonogenic ability compared with either CXCR2 or CXCR3 knockdown alone. Moreover, CXCR2 and CXCR3 silencing significantly reduced the sphere‐forming ability of RCC cells. High expression levels of CXCR2 and CXCR3 in cancer tissues correlated with tumour progression of renal cell carcinoma. These findings suggest that TNF‐α augments CXCR2 and CXCR3 to promote the progression of renal cell carcinoma leading to a poor prognosis.

## Introduction

Renal cell carcinoma (RCC) is the most common type of kidney cancer in adults. Surgery can cure localized disease, however, many patients experience recurrence after surgery or have metastatic disease at the time of diagnosis 1. RCC has a highly resistant phenotype to conventional chemotherapy and radiation. Improved understanding of tumour biology has led to the development of novel targeted therapeutic agents that have modestly improved the prognosis of such patients, however, cancer recurrence due to drug resistance is still a critical issue 2. The development of more effective and highly selective molecular targeting agents is therefore important to improve the current treatment for RCC.

The tumour microenvironment consists of immune cells, stromal cells, extracellular matrix and neovasculature. It forms a complex regulatory network that fosters tumour growth by evading immune surveillance and destruction 3, 4, 5. In addition, an inflammatory tumour microenvironment promotes epithelial‐mesenchymal transition (EMT), angiogenesis and the formation of cancer stem cells, which contribute to tumour initiation, tumour progression, metastasis and resistance to therapy 5, 6. It is known that tumours and stroma interact through a variety of cytokines, chemokines, and growth factors. The main inflammatory cytokines, for example, tumour necrosis factor‐alpha (TNF‐α), interleukin‐6 (IL‐6) and IL‐8; chemokines such as chemokine (C‐X‐C Motif) Ligand 12 (CXCL12); and growth factors, for instance, vascular endothelial growth factor (VEGF), are crucial factors present in the inflammatory tumour microenvironment 6.

Chemokines are produced by tumour cells, stromal cells and tumour‐associated leucocytes. They are potent attractants of leucocytes, such as neutrophils, monocytes, natural killer cells and T cells. The target cell specificity of each chemokine is determined by the expression of its cognate receptor. Chemokines are structurally divided into four subgroups of CXC, CC, CX3C and C; and are functionally classified as inflammatory, homoeostatic or both 7. The CXC chemokines with an amino acid sequence of glutamic acid‐leucine‐arginine (the ELR motif) are the typical inflammatory and angiogenic chemokines 7, 8. The binding of ELR^+^ CXC chemokines to CXCR2 will promote neovascularization 8; whereas the binding of ELR^−^ CXC chemokines to CXCR3 is angiostatic 9. One angiogenic exception of the ELR^−^ CXC chemokines is CXCL12. CXCR4 is the corresponding receptor of CXCL12 and is commonly overexpressed in many human cancers, including RCC. In addition to CXCR4, CXCR7 and CXCL5 are thought to be important regulators of the survival, growth, and metastasis of RCC cells 10, 11, 12, 13.

TNF‐α, a major mediator of cancer‐related inflammation in the tumour environment, can induce the generation of various types of mediators, including reactive oxygen intermediates, cyclooxygenase, matrix metalloproteinases (MMPs) and cytokines 14, 15. The chronic low dose of TNF‐α produced by a number of cancer cells, including RCC, and stromal cells may promote tumour growth and metastasis. Furthermore, the presence of TNF‐α in tumours has been associated with a poor prognosis, hormone resistance and cachexia 14, 15. The serum levels of TNF‐α have also been reported to be significantly higher and correlated with tumour size in patients with RCC 16, 17. In addition, infliximab, a chimeric anti‐TNF‐α monoclonal antibody, has successfully been used to treat RCC patients in phase II clinical studies 18. TNF‐α has also been shown to induce EMT and promote tumorigenicity in RCC cells 19, 20, 21, 22. However, the role of CXC chemokines and their receptors in the TNF‐α‐promoted progression of RCC remain unclear. In this study, TNF‐α was found to enhance the migration, invasion and EMT of RCC cells. To further investigate the molecular mechanism of TNF‐α on the progression of RCC, we used reverse transcription and quantitative PCR (RT‐qPCR) to detect chemokines and chemokine receptors that were associated with tumorigenesis. The results showed that TNF‐α significantly increased the expressions of CXCR2 and CXCR3 and their related ligands in RCC cells. These findings suggest a novel molecular mechanism in which TNF‐α augments CXCR2 and CXCR3 to promote the progression of RCC resulting in a poor prognosis.

## Materials and methods

### Cell lines

Human RCC cell lines 786‐O (ATCC no. CRL‐1932), A‐498 (ATCC no. HTB‐44), Caki‐1 (ATCC no. HTB‐46) and ACHN (ATCC no. CRL‐1611) were purchased from BCRC (Bioresource Collection and Research Center; Hsinchu, Taiwan) and ATCC (American Type Culture Collection, USA).

### Wound healing assay

The 786‐O and A‐498 cells (7 × 10^5^ in 3 ml) were cultured overnight to reach at least 80% confluence and scratched with a pipette tip to create a wound area. The cells were then treated with or without TNF‐α (50 ng/ml, Invitrogen, Carlsbad, CA, USA) for the indicated time. Cell migration images were obtained and cell mobility was quantified using Image J software (NIH, Bethesda, MD, USA). Wound healing rate was quantified as distant cells migrated across the injury line during incubation.

### Invasion assay

The 786‐O and A498 RCC cells (2 × 10^5^ in 3 ml) were cultured overnight and then treated with or without TNF‐α (50 ng/ml). After 3 days of culture, the invasive ability of RCC cells was assessed by Transwell assay (Costar, 8‐μm pore; Corning, NY, USA), and each insert was coated with Matrigel (BD Biosciences, San Jose, CA, USA). RCC cells (2 × 10^4^) were resuspended in 100 μl 0.5% foetal bovine serum (FBS)‐cultured medium and plated in the upper chamber. The lower chamber was filled with 800 μl 10% FBS‐cultured medium. After incubation for 20 hrs at 37°C, the cells that had invaded on the lower surfaces of the inserts were fixed with methanol. The Matrigel and non‐penetrating cells in the upper chamber were mechanically wiped using cotton swabs. Subsequently, the chambers were stained with Liu's stain (Muto Pure Chemical, Tokyo, Japan) and counted under light microscopy (×100 magnifications).

### Reverse transcription and quantitative PCR

RCC cells (6 × 10^5^ in 3 ml) were cultured overnight and then treated with or without TNF‐α (50 ng/ml) for 24 hrs. TRIzol reagent (Invitrogen) was applied to extract total cellular RNA. Five μg of RNA was used to synthesize cDNA using a SuperScript^®^ III First‐Strand Synthesis System (Invitrogen). Quantitative RT‐PCR was performed with Fast SYBR Green Master Mix (Applied Biosystems, Foster City, CA, USA) using an ABI7700 System (Applied Biosystems). Values were normalized against the mRNA level of glyceraldehyde‐3‐phosphate dehydrogenase (GAPDH) to obtain the ΔCt. Then, the relative expression to control was determined by subtracting the ΔCt of the experimental sample from the ΔCt of the control sample. The specific primers used in the RT‐qPCR are described in Table S1.

### Western blot

The 786‐O and A498 RCC cells (2 × 10^5^ in 3 ml) were cultured overnight and then treated with or without TNF‐α (50 ng/ml) for 4 days. Equal amounts (60 μg) of total cellular proteins were separated on 10% SDS‐PAGE, electroblotted onto a nitrocellulose (NC) membrane (Millipore, Temecula, CA, USA), probed with anti‐E‐cadherin (BD Biosciences), anti‐vimentin (Sigma‐Aldrich, St Louis, MO, USA), anti‐GAPDH (Sigma‐Aldrich), anti‐CXCR2 or anti‐CXCR3 monoclonal antibodies (R&D Systems, Minneapolis, MN, USA) and horseradish peroxidase‐conjugated secondary antibodies, and then analysed using enhanced chemiluminescence.

### Flow cytometry

The A498 cells (8 × 10^5^ in 10 ml) were cultured overnight and then treated with or without TNF‐α (50 ng/ml). After 3 days of culture, cells (1 × 10^6^) were stained with anti‐CXCR2 or anti‐CXCR3 monoclonal antibodies (R&D Systems) for 1 hour at 4°C. The cells were then stained with fluorescein isothiocyanate (FITC)‐anti‐mouse‐IgG for 30 min. at 4°C, resuspended in 1 ml PBS and analysed by flow cytometry (BD FACSCalibur: BD Bioscience, San Jose, CA, USA).

### Lentivirus‐mediated knockdown of CXCR2 and CXCR3

Lentivirus‐mediated silencing of CXCR2 and CXCR3 of the A498 cells was performed as in our previous study 23. The pLKO.1 plasmid containing shRNA targeting human CXCR2 (shCXCR2) or CXCR3 (shCXCR3) was purchased from the National RNAi Core Facility (Academia Sinica, Taipei, Taiwan). The specific target sequences for shCXCR2#6 and shCXCR3#17 were 5′‐CCGTCTACTCATCCAATGTTA‐3′ (Clone ID TRCN0000009138) and 5′‐CCGCTGCTCTATGCCTTTGTA‐3′ (Clone ID TRCN0000011317) respectively. A control vector expressing shRNA against luciferase (pLKO.1‐shLuc) was used as a negative control.

### Clonogenic assay

The clonogenic assay determines the ability of a cell to proliferate indefinitely to form a large colony. The A498 cells (1 × 10^2^) were plated in six‐well plates and incubated with or without TNF‐α (25 ng/ml) for 10 days. The cells were then fixed with methanol, stained with 0.5% crystal violet and the colonies were counted.

### Sphere formation assay

To enrich the kidney cancer stem cells, A498 cells (2 × 10^2^/200 μl) were cultured in tumour sphere medium containing serum‐free Dulbecco's modified eagle medium (DMEM)/F12 (1:1) medium, 1X B27 supplement, 20 ng/ml human recombinant basic fibroblast growth factor, and 20 ng/ml epidermal growth factor (Gibco, BRL, Life Tech.: Grand Island, New York, USA). A‐498 cells were incubated with medium that was replaced every 3–4 days and cultured for a total of 20 days. The number of spheres was counted using a microscope.

### Immunohistochemistry

Tissue microarray (TMA) slides were bought from Biomax (US Biomax Inc., Rockville, MD, USA). After deparaffinizing, rehydrating, heat‐induced epitope retrieval and blocking with 3% H_2_O_2_, the treated TMA slides were incubated with CXCR2 and CXCR3 (R&D Systems) primary antibody and the immunohistochemistry was performed as described previously 23. The slides were analysed using the Aperio ImageScope (Aperio Technologies Inc., Vista, CA, USA) and a digital stained cell score was obtained. The detail clinicopathologic characteristics of the patients included in the TMA are listed in Table S2.

### Statistical analysis

The results were presented as the mean ± standard deviation (SD). Differences between two groups were examined using the Student's *t*‐test. Gene expression relevance between different stages of RCC was analysed by non‐parametric Mann–Whitney *U*‐test.

Gene expression of CXCR2 and CXCR3 in clear cell RCC were analysed using the SurvExpress web‐based tool 24. By Cox survival analysis, a population of kidney renal clear cell carcinoma patients (accession no. TCGA) was classified into high‐ and low‐risk groups according to their genetic profiles on the basis of survival (prognostic index).

## Results

### TNF‐α enhanced migration, invasion and EMT of RCC cells

**Figure 1:**
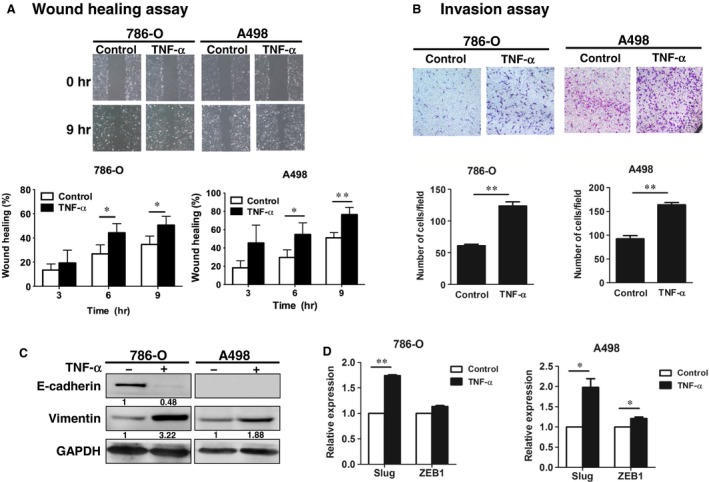
TNF‐α enhanced migration, invasion and EMT of RCC cells. (**A**) 780‐O and A498 cells were scratched and treated with or without TNF‐α (50 ng/ml) for the indicated times. Cell migration was quantified. (**B**) 786‐O and A498 cells were treated with or without TNF‐α (50 ng/ml) for 3 days, and the invasion ability was examined. (**C**,** D**)*,* 786‐O and A498 cells were treated with or without TNF‐α (50 ng/ml), and the EMT markers were examined by Western blot (**C**) and RT‐qPCR (**D**). The results are representative of three independent experiments. *P < 0.05; **P < 0.01. EMT, epithelial‐mesenchymal transition; TNF‐α, tumour necrosis factor‐alpha.

EMT is the process where epithelial cells lose cell polarity and cell–cell adhesion and become mesenchymal cells with migratory and invasive properties. This process plays a crucial role in the initiation of metastasis in cancer progression. Therefore, we first examined whether TNF‐α promoted the metastatic ability of RCC cells. The wound healing assays were used to measure tumour cell migration ability. The wound healing assay showed that TNF‐α time‐dependently enhanced the migration of 786‐O and A498 RCC cells (Fig. 1A). In addition, the invasive ability of RCC cells assessed by Matrigel‐coated Transwell was significantly promoted by TNF‐α after 3 days of treatment (Fig. 1B). Furthermore, the mesenchymal marker vimentin (Fig. 1C) and the mRNA levels of the EMT‐associated transcriptional factors Slug and ZEB1 in A498 cells (Fig. 1D) were significantly up‐regulated in response to TNF‐α. E‐cadherin, an epithelial marker, was decreased in TNF‐α‐treated 780‐O cells. Therefore, TNF‐α promoted the motility of RCC cells.

### TNF‐α increased expressions of CXC chemokines and receptors in RCC cells

**Figure 2:**
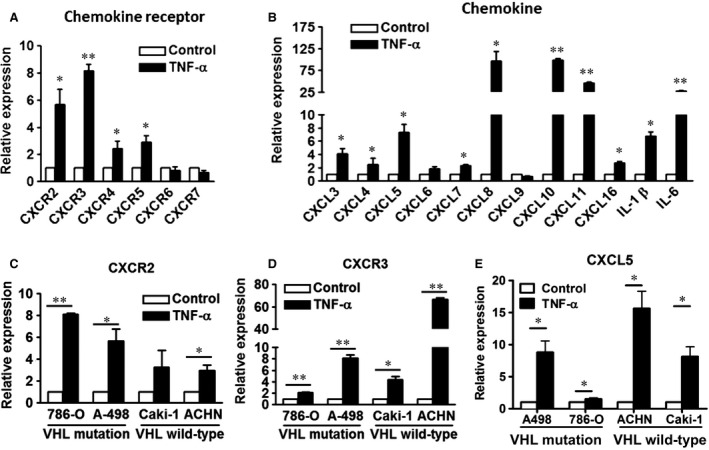
TNF‐α increased the expressions of CXC chemokines and receptors in RCC cells. A498 (**A**,** B**) and RCC cells (**C**,** D**,** E**) were treated with or without TNF‐α (50 ng/ml) for 24 hrs. The expression levels of chemokine receptors (**A**), chemokines (**B**), CXCR2 (**C**), CXCR3 (**D**) and CXCL5 (**E**) were examined by RT‐qPCR. The results are representative of three independent experiments. *P < 0.05; **P < 0.01. TNF‐α, tumour necrosis factor‐alpha.

**Figure 3:**
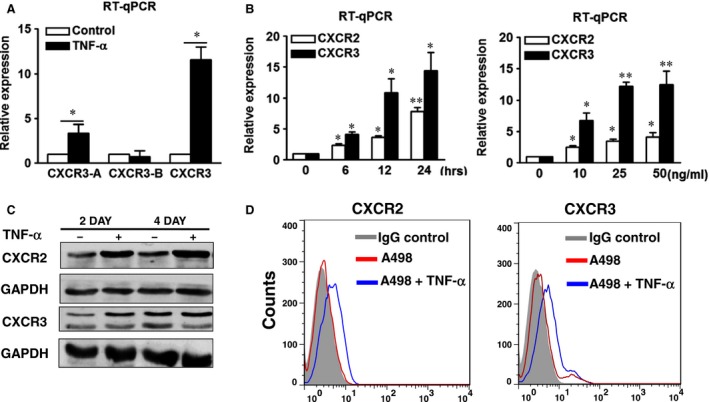
TNF‐α increased the expressions of CXCR2 and CXCR3 in A498 cells. A498 cells were treated with or without TNF‐α (50 ng/ml) for 24 hrs (**A**), or different times and different doses (**B**). The expression levels of CXCR2, CXCR3‐A, CXCR3‐B and CXCR3 were examined by RT‐qPCR (**A**,** B**), Western blot (**C**) and flow cytometry (**D**). The results are representative of three independent experiments. GAPDH, glyceraldehyde‐3‐phosphate dehydrogenase. *P < 0.05; **P < 0.01. TNF‐α, tumour necrosis factor‐alpha.

To investigate the role of the CXC chemokine system in the TNF‐α‐promoted motility of RCC, RT‐qPCR was used to detect the chemokines and receptors that were affected by TNF‐α. As shown in Figure 2A and B, TNF‐α highly increased the expressions of CXCR2 and CXCR3 and their related ligands (CXCL3, CXCL4, CXCL5, CXCL7, CXCL8, CXCL10 and CXCL11) in A498 cells. Among these ligands, CXCL8, CXCL10 and CXCL11 were robustly enhanced by TNF‐α. In addition, CXCR4, CXCR5, CXCL16, IL‐1β and IL‐6 were also significantly up‐regulated by TNF‐α. However, TNF‐α did not promote expressions of CXCR6, CXCR7, CXCL6 and CXCL9 in A498 cells. Moreover, the TNF‐α‐enhanced expressions of CXCR2 (Fig. 2C), CXCR3 (Fig. 2D) and CXCL5 (Fig. 2E) were confirmed in von Hippel‐Lindau (VHL) wild‐type and mutated RCC cells. Since CXCR3 has two splice variants, CXCR3‐A and CXCR3‐B, with growth‐promotion and growth‐inhibition activities respectively 25, we examined the effect of TNF‐α on the RNA expressions of these two variants in A498 cells. In contrast to CXCR3‐B, TNF‐α significantly increased CXCR3‐A expression (Fig. 3A).

To further verify the effect of TNF‐α on the expressions of CXCR2 and CXCR3, A498 cells were treated with TNF‐α for different durations and doses. TNF‐α gradually stimulated the RNA expressions of CXCR2 and CXCR3 in A‐498 cells for 6–24 hrs (Fig. 3B, left panel). In addition, TNF‐α enhanced the expressions of CXCR2 and CXCR3 in a dose‐dependent manner (Fig. 3B, right panel). The TNF‐α‐induced increased levels of CXCR2 and CXCR3 proteins in A498 cells were confirmed by Western blot (Fig. 3C) and flow cytometry (Fig. 3D). The results indicated that TNF‐α promoted the expressions of CXCR2 and CXCR3 in RCC cells.

### CXCR2 and CXCR3 knockdown inhibited migration, invasion, clonogenic and sphere‐forming abilities of RCC cells

**Figure 4:**
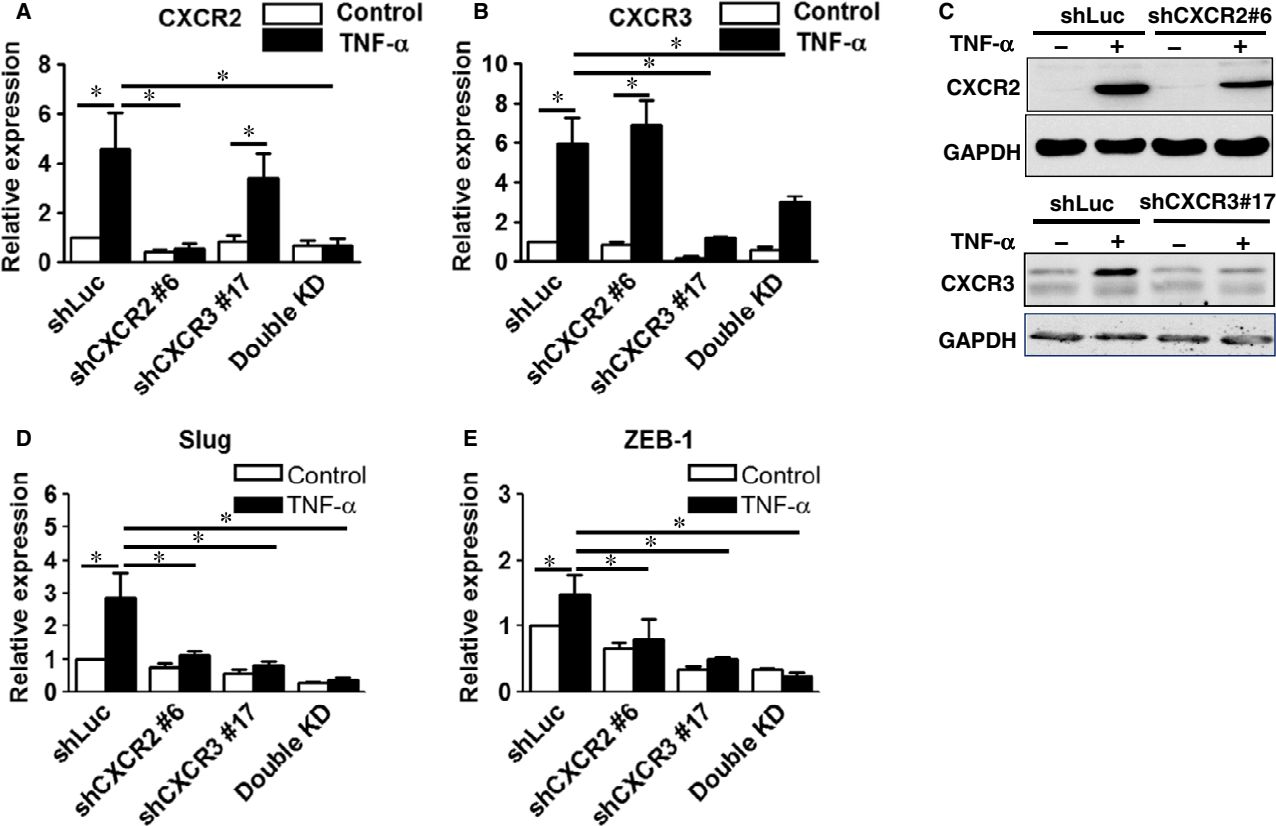
Knockdown of CXCR2 and CXCR3 were confirmed and CXCR2 and CXCR3 silencing decreased the expressions of Slug and ZEB‐1 in A498 cells. Knockdown of CXCR2 and CXCR3 and CXCR2/CXCR3 (Double knockdown) in A498 cells was confirmed by RT‐qPCR (**A**,** B**) and Western blot (**C**) after treating with or without tumour necrosis factor‐alpha. (50 ng/ml). Expression levels of Slug and ZEB‐1 in the silenced cells were examined by RT‐qPCR (**D**,** E**). The results are representative of three independent experiments. *P < 0.05, **P < 0.05.

**Figure 5:**
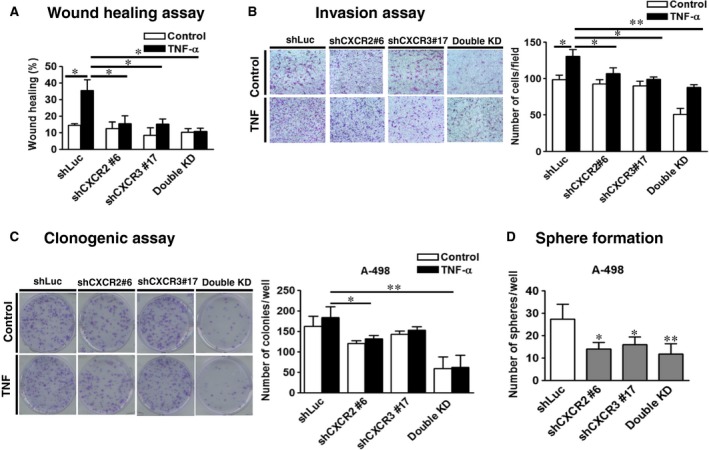
CXCR2 and CXCR3 knockdown inhibited the migration, invasion, clonogenic and sphere‐forming ability of A498 cells. Wound healing assay (**A**), invasion assay (**B**) and clonogenic assay (**C**) were performed in various knocked down A498 cells after treating with or without tumour necrosis factor‐alpha. (50 ng/ml). (**D**) Sphere‐forming abilities were analysed in shLuc‐, shCXCR2‐, shCXCR3‐ and shCXCR2/shCXCR3 (Double knockdown)‐infected A498 cells. Cells (200 cells/well) were cultured in tumour sphere medium for 20 days. The results are representative of three independent experiments. *P < 0.05; **P < 0.01.

To explore the role of CXCR2 and CXCR3 in the TNF‐α‐promoted tumorigenesis of RCC, CXCR2 and CXCR3‐A were silenced using a lentiviral shRNA system in A498 cells. Eight clones of shCXCR2 and nine clones of shCXCR3 from the RNAi core facility were screened for their silencing effects on the expressions of CXCR2 and CXCR3 in A498 cells (data not shown). Compared to the shLuc control, shCXCR2#6 and/or shCXCR3#17 efficiently silenced the RNA and protein expressions of CXCR2 (Fig. 4A and C) and CXCR3 (Fig. 4B and C) induced by TNF‐α in single and double knockdown (KD) cells. In addition, CXCR2 and/or CXCR3 single and double knockdown significantly decreased the mRNA levels of the EMT‐associated transcriptional factors Slug (Fig. 4D) and ZEB‐1 (Fig. 4E) promoted by TNF‐α in A498 cells. CXCR2 or CXCR3 single knockdown can significantly inhibit the cell migration (Fig. 5A), invasion (Fig. 5B), and clonogenic abilities (Fig. 5C) promoted by TNF‐α. Knockdown of both CXCR2 and CXCR3 (double KD) resulted in a greater decrease, compared with single knockdown. These results indicated that CXCR2 and CXCR3 silencing downregulated the metastatic and growth ability of RCC cells.

In addition to invasion and EMT, acquired stem cell‐like properties of cancer cells are involved in metastasis and drug resistance. The sphere formation assay is widely used to assess the self‐renewal potential of stem‐like cancer cells 26. Control and knockdown A498 cells were seeded at clonal density (200 cells/well) and cultured for 20 days. CXCR2 and/or CXCR3 silencing significantly reduced the sphere‐forming ability of RCC cells (Fig. 5D).

### High expression levels of CXCR2 and CXCR3 in cancer tissues correlated with tumour progression of renal clear cell carcinoma

**Figure 6:**
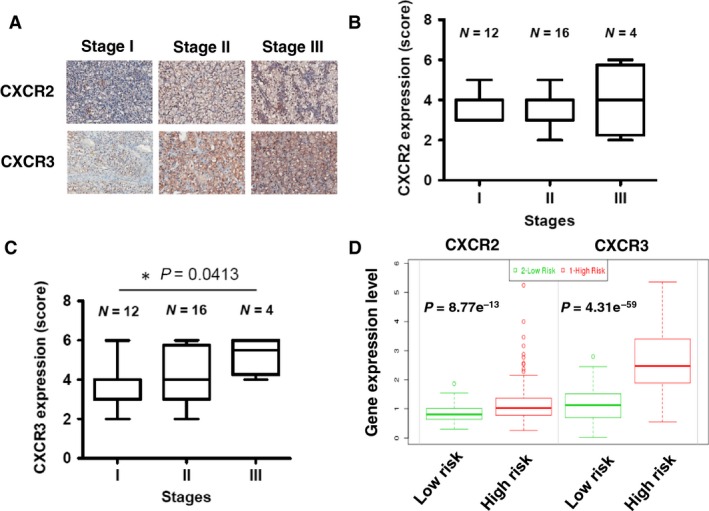
CXCR2 and CXCR3 expression correlated with tumour progression in tissues of clear cell renal cell carcinoma (ccRCC). (**A**) Immunohistochemistry staining of CXCR2 and CXCR3 was performed on tissue microarrays containing samples from 32 patients with ccRCC. (**B**) CXCR2 protein expression levels were quantified in 32 tissues of different stages of ccRCC. (**C**) CXCR3 protein expression levels were quantified in 32 tissues of different stages of ccRCC. (**D**) CXCR2 and CXCR3 gene expression between risk groups in ccRCC (accession no. TCGA) using the SurvExpress web‐based analysis (http://bioinformatica.mty.itesm.mx/SurvExpress). *P < 0.05.

To further study, the association between CXCR2/CXCR3 expression and tumour progression in patients, the commercial RCC TMA and immunohistochemistry were used (Fig. 6A). High expression levels of CXCR2 or CXCR3 were found to be associated with patients with advanced stage renal clear cell carcinoma (Fig. 6B and C). In addition, we used the online tool, SurvExpress 24, to further analyse the association between CXCR2/CXCR3 expression and patient prognosis. By Cox survival analysis, risk estimation was performed in 468 patients with different stages of renal clear cell carcinoma (accession no. TCGA) using the prognostic index. High expression levels of CXCR2 or CXCR3 were found to be significantly associated with the high‐risk group of patients with renal clear cell carcinoma (Fig. 6D).

## Discussion

Chronic inflammation in the tumour microenvironment plays a crucial role in tumorigenesis. Inflammatory cytokines and chemokines in tumour microenvironments recruit mesenchymal stem cells (MSCs) into tumour tissues to educate these MSCs to promote tumour growth 6. Tumour MSCs can attract immunosuppressive cells, and enhance EMT, tumour angiogenesis and cancer stem cell formation. The acquisition of stem‐like properties of cancer cells is involved in metastasis and drug resistance. TNF‐α is a master switch from chronic inflammation to cancer 14, 15. As higher levels of TNF‐α are associated with advanced RCC 16, 17, we investigated the molecules involved in the TNF‐α‐promoted progression of RCC. Our results showed that CXCR2 and CXCR3 were the most induced chemokine receptors in response to TNF‐α.

While the induction of CXCR2 and ligands by Kras has been shown to reinforce senescence *in vitro* and is thought to be protective in the early stages of tumorigenesis 27, CXCR2 ligands have been implicated in the angiogenesis and proliferation of tumours and in neutrophil recruitment to the tumour 7, 8. The overexpression of CXCR2 and its ligands has been noted in many cancers, and it has been reported to be involved in tumour growth and development 28, 29. The CXCR2 ligands CXCL1, CXCL3, CXCL5 and CXCL8 have also been reported to be elevated in the plasma of RCC patients, with CXCR2 being expressed on endothelial cells in RCC tissues 11. Furthermore, RCC cell lines and fresh tumours express CXCR2, and the specific inhibitor of CXCR2 inhibits the proliferation of RCC cells *in vitro* 30. The blockade of CXCR2 has been shown to reduce tumour growth and angiogenesis in mice with RCC 30. These findings suggest the importance of CXCR2 in the progression of RCC.

Emerging evidence suggests that the CXCR3 signalling network can positively influence tumour cell growth and metastasis 7. CXCR3 and its ligands are expressed in many human cancers, and it is considered to be a poor prognostic factor 31, 32, 33. Moreover, in a murine model, antagonism of CXCR3 by a small molecule inhibitor blocked pulmonary metastasis of breast cancers 32. Only a few studies have investigated the relationship between RCC and CXCR3. Two reports showed that the expression of CXCR3 or its ligands were related to a good prognosis in patients with localized RCC 34, 35. Conversely, Utsumi *et al*. demonstrated an association between CXCR3 expression with RCC metastasis, and they reported that hypoxia may induce the expression of CXCR3 36. However, determining the role of CXCR3 in tumorigenesis is complicated by the fact that many cells in the tumour microenvironment potentially express CXCR3 splice variants and their ligands. In human RCC tissues, the expression of growth‐promoter CXCR3‐A is increased, and that of growth‐inhibitor CXCR3‐B decreased 25. Furthermore, it has been demonstrated that CXCR3‐B promotes mammosphere formation 37. In this study, TNF‐α augmented the expressions of CXCR3 and its ligands, and the knockdown of CXCR3‐A downregulated the EMT and sphere formation ability of RCC cells.

Immunotherapy therapy with interferon‐alpha (IFN‐α) and interleukin‐2 (IL‐2) is the standard treatment for metastatic RCC. In addition, several molecule targeting drugs, including tyrosine kinase inhibitors, mTOR inhibitors and monoclonal antibodies against VEGF, have been used for advanced RCC 1, 2. However, most patients acquire drug resistance at around 6–11 months. Expression of TNF‐α and CD44 cancer stem cell marker is implicated in the drug resistance of RCC patients 17. Chemotherapy‐induced CXC chemokine/receptor also confers drug resistance by promoting cancer stem cell formation 38, 39. Therefore, the tumour microenvironment is changed dynamically before and after therapy. Monitoring the status of the tumour microenvironment is important for precision medicine, and targeting the tumour microenvironment is a crucial adjunct to the standard therapy of cancers. The result of present study seems feasible to provide a novel molecular mechanism to improve and optimize the treatment of patients with advanced RCC.

In conclusion, this study is the first to demonstrate that TNF‐α, a key mediator in the inflammatory tumour microenvironment, strongly up‐regulated CXCR2 and CXCR3 to enhance migration, invasion, EMT and sphere formation of RCC cells. RCC patients with high expression levels of CXCR2 and CXCR3 had a significantly worse prognosis. Thus, the TNF‐α/CXCR2/CXCR3 axis may be a prognostic marker and provide a novel target for combination therapies for advanced RCC in the future.

## Conflict of interest

There is no conflict of interest.

## Supporting information


**Table S1** Primer sequences used in RT‐qPCR.

**Table S2** Clinicopathologic characteristics of patients with kidney cancer included in this study.
