# Supplementary material for: TNF‐α augments CXCR2 and CXCR3 to promote progression of renal cell carcinoma
Source: J Cell Mol Med. 2016 Jun 14;20(11):2020–8. doi: 10.1111/jcmm.12890 (PMC5082409; doi:10.1111/jcmm.12890)
Supplement: Supplementary file 1 — Table S1 Primer sequences used in RT‐qPCR. [file JCMM-20-2020-s001.docx]

**Table S1.** Primer sequences used in RT-qPCR

| **Genes** | **Forward primer/Reverse primer** |
| --- | --- |
| CXCL3 | 5’-TGGTCACTGAACTGCGCT-3’  5’-ATGCGGGGTTGAGACAAG-3’ |
| CXCL4 | 5’-TGAAGAATGGAAGGAAAATTTGC-3’  5’-CAAATGCACACACGTAGGCAGCT-3’ |
| CXCL5 | 5’-AGAGCTGCGTTGCGTTTGT-3’  5’-CTATGGCGAACACTTGCAGATTAC-3’ |
| CXCL6 | 5’-AGAGCTGCGTTGCACTTGTT-3’  5’-GCAGTTTACCAATCGTTTTGGGG-3’ |
| CXCL7 | 5’-GAACTCCGCTGCATGTGTATAAAG-3’  5’-GCATCTGGGTCCAGGCAG-3’ |
| CXCL8 | 5’-ACTGAGAGTGATTGAGAGTGGACC-3’  5’-ACAACCCTCTGCACCCAGTT-3’ |
| CXCL9 | 5’-CCAGTAGTGAGAAAGGGTCGC-3’  5’-AGGGCTTGGGGCAAATTGTT-3’ |
| CXCL10 | 5’-AAGCCAATTTTGTCCACGTGTT-3’  5’-TGGCCTTCGATTCTGGATTC-3’ |
| CXCL11 | 5’-GACGCTGTCTTTGCATAGGC-3’  5’-GGATTTAGGCATCGTTGTCCTTT-3’ |
| CXCL16 | 5’-GGCCCACCAGAAGCATTTAC-3’  5’-CTGAAGATGCCCCCTCTGAG-3’ |
| CXCR2 | 5’-CATGGCTTGATCAGCAAGGA-3’  5’-TGGAAGTGTGCCCTGAAGAAG-3’ |
| CXCR3 | 5’-CAGGTGCCCTCTTCAACATCA-3’  5’-ATGTTCAGGTAGCGGTCAAAGC-3’ |
| CXCR3-A | 5’-GGTCCTTGAGGTGAGT-3’  5’-GCTGAAGTCCTGTGGG-3’ |
| CXCR3-B | 5’-TGCCAGGCCTTTACACAGC-3’  5’-TCGGCGTCATTTAGCACTTG-3’ |
| CXCR4 | 5’-CGTCAGTGAGGCAGATGAC-3’  5’-TGCAATAGCAGGACAGGATG-3’ |
| CXCR5 | 5’-GGTCACCCTACCACATCGTC-3’  5’-GCCATTCAGCTTGCAGGTATTG-3’ |
| CXCR6 | 5’-GACTATGGGTTCAGCAGTTTCA-3’  5’-GGCTCTGCAACTTATGGTAGAAG-3’ |
| CXCR7 | 5’-GGCTATGACACGCACTGCTACA-3’  5’-TGGTTGTGCTGCAC-3’ |
| IL-1β | 5’-TAAAGCCCGCCTGACAGAA-3’  5’-GGAGCGAATGACAGAGGGTTT-3’ |
| IL-6 | 5’-CACCGGGAACGAAAGAGAAG-3’  5’-CGCTTGTGGAGAAGGAGTTCAT-3’ |
| vimentin | 5’-CAGCATCACGATGACC-3’  5’-TCTTGCGCTCCTGAAA-3’ |
| Slug | 5’-TGTTGCAGTGAGGGCAAGAA-3’  5’-GACCCTGGTTGCTTCAAGGA-3’ |
| ZEB-1 | 5’-GCCAATAAGCAAACGATTCTG-3’  5’-TTTGGCTGGATCACTTTCAAG-3’ |
| GAPDH | 5’-CAACTACATGGTTTACATGTTC-3’  5’-GCCAGTGGACTCCACGAC-3’ |
