# Supplementary material for: TNF‐α augments CXCR2 and CXCR3 to promote progression of renal cell carcinoma
Source: J Cell Mol Med. 2016 Jun 14;20(11):2020–8. doi: 10.1111/jcmm.12890 (PMC5082409; doi:10.1111/jcmm.12890)
Supplement: Supplementary file 2 — Table S2 Clinicopathologic characteristics of patients with kidney cancer included in this study. [file JCMM-20-2020-s002.docx]

Table S2. Clinicopathologic characteristics of patients with kidney cancer included in this study

| **Case** | **Age** | **Sex** | **Pathology** | **Grade** | **Stage** | **TNM** |
| --- | --- | --- | --- | --- | --- | --- |
| **1** | 48 | M | Chromophobe carcinoma | - | II | T2N0M0 |
| **2** | 52 | M | Chromophobe carcinoma | - | II | T2N0M0 |
| **3** | 77 | M | Chromophobe carcinoma | - | II | T2N0M0 |
| **4** | 72 | M | Chromophobe carcinoma | - | III | T2N1M0 |
| **5** | 45 | F | Clear cell carcinoma | 1 | I | T1N0M0 |
| **6** | 53 | F | Clear cell carcinoma | 1 | I | T1N0M0 |
| **7** | 59 | F | Clear cell carcinoma | 1 | I | T1N0M0 |
| **8** | 64 | F | Clear cell carcinoma | 1 | I | T1N0M0 |
| **9** | 46 | M | Clear cell carcinoma | 1 | I | T1N0M0 |
| **10** | 59 | M | Clear cell carcinoma | 1 | I | T1N0M0 |
| **11** | 67 | M | Clear cell carcinoma | 1 | I | T1N0M0 |
| **12** | 67 | M | Clear cell carcinoma | 1 | I | T1N0M0 |
| **13** | 70 | M | Clear cell carcinoma | 1 | I | T1N0M0 |
| **14** | 74 | M | Clear cell carcinoma | 1 | I | T1N0M0 |
| **15** | 61 | M | Clear cell carcinoma | 2 | I | T1N0M0 |
| **16** | 67 | M | Clear cell carcinoma | 2 | I | T1N0M0 |
| **17** | 33 | F | Clear cell carcinoma | 1 | II | T2N0M0 |
| **18** | 40 | F | Clear cell carcinoma | 1 | II | T2N0M0 |
| **19** | 49 | F | Clear cell carcinoma | 1 | II | T2N0M0 |
| **20** | 26 | M | Clear cell carcinoma | 1 | II | T2N0M0 |
| **21** | 48 | M | Clear cell carcinoma | 1 | II | T2N0M0 |
| **22** | 49 | M | Clear cell carcinoma | 1 | II | T2N0M0 |
| **23** | 57 | M | Clear cell carcinoma | 1 | II | T2N0M0 |
| **24** | 38 | F | Clear cell carcinoma | 2 | II | T2N0M0 |
| **25** | 61 | F | Clear cell carcinoma | 2 | II | T2N0M0 |
| **26** | 39 | M | Clear cell carcinoma | 2 | II | T2N0M0 |
| **27** | 49 | M | Clear cell carcinoma | 2 | II | T2N0M0 |
| **28** | 52 | M | Clear cell carcinoma | 2 | II | T2N0M0 |
| **29** | 59 | M | Clear cell carcinoma | 2 | II | T2N0M0 |
| **30** | 63 | M | Clear cell carcinoma | 2 | II | T2N0M0 |
| **31** | 65 | M | Clear cell carcinoma | 2 | II | T2N0M0 |
| **32** | 50 | F | Clear cell carcinoma | 1 | IV | T4N1M0 |
| **33** | 63 | F | Clear cell carcinoma | 1 | III | T2N1M0 |
| **34** | 80 | M | Clear cell carcinoma | 1 | III | T3aN0M0 |
| **35** | 45 | M | Clear cell carcinoma | 2 | III | T3aN0M0 |
| **36** | 46 | F | Clear cell carcinoma | 2 | III | T3bN0M0 |
| **37** | 30 | F | Clear cell carcinoma | 2 | II | T2bN0M0 |
| **38** | 39 | F | Papillary renal cell carcinoma | - | I | T1N0M0 |
| **39** | 18 | M | Papillary renal cell carcinoma | - | I | T1N0M0 |
| **40** | 46 | M | Papillary renal cell carcinoma | - | I | T1N0M0 |
| **41** | 47 | M | Papillary renal cell carcinoma | - | I | T1N0M0 |
| **42** | 48 | M | Papillary renal cell carcinoma | - | I | T1N0M0 |
| **43** | 49 | M | Papillary renal cell carcinoma | - | I | T1N0M0 |
| **44** | 62 | M | Papillary renal cell carcinoma | - | I | T1N0M0 |
| **45** | 70 | M | Papillary renal cell carcinoma | - | II | T2N0M0 |
| **46** | 35 | M | Papillary renal cell carcinoma | - | II | T1N1M0 |
